# Independent respiratory navigators for improved 3D PSIR imaging of myocardial infarctions

**DOI:** 10.1186/1532-429X-13-S1-P18

**Published:** 2011-02-02

**Authors:** Sangjune L Lee, Michael Schär, Sebastian Kozerke, Ahmed A Harouni, Valeria Sena-Weltin, M Muz Zviman, Henry Halperin, Elliot R McVeigh, Daniel A Herzka

**Affiliations:** 1 Department of Biomedical Engineering, Johns Hopkins University School of Medicine, Baltimore, MD, USA; 2 Philips Healthcare, Cleveland, OH, USA; 3 Institute for Biomedical Engineering, University and ETH Zurich, Zurich, Switzerland; 4 Department of Electrical and Computer Engineering, Johns Hopkins University, Baltimore, MD, USA; 5 Division of Cardiology, Johns Hopkins University School of Medicine, Baltimore, MD, USA

## Introduction

**Figure 1:**
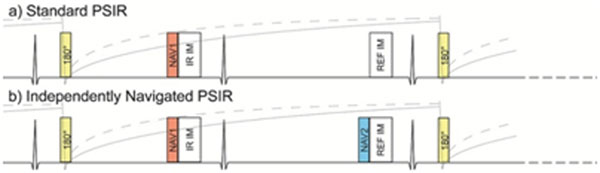
Schematic of PSIR and INPSIR acquisitions. The second independent navigator provides motion compensation for the phase reference images.

The distribution of viable and infarcted myocardium is typically visualized using inversion recovery (IR) late gadolinium enhancement (1) or phase-sensitive inversion recovery (PSIR) sequences (2). Transitioning PSIR from breath-hold 2D to respiratory navigator-gated 3D imaging promises higher SNR and CNR, and whole heart coverage (3,4). However, the optimal method for motion compensation with 3D PSIR is undetermined. With standard PSIR, the IR-prepared volume (first heartbeat) is corrected with the phase from the reference volume (second heartbeat), (Fig 1a). Current implementations of respiratory navigated 3D PSIR accept data for the reference based solely on navigator NAV1, which takes place over a heartbeat in advance. Respiratory motion occuring between NAV1 and reference volume acquisition, potentially corrupts reference image quality and may compromise the PSIR image. We propose an independently navigated PSIR (INPSIR) sequence with a separate navigator, NAV2, dedicated to motion compensation of the reference volume (Fig 1b).

## Methods

Imaging was performed on a 3T system (Philips Healthcare, Best, The Netherlands) using a 32-channel cardiac array coil (InVivo, Gainsville FL). Phantoms: agar gel phantoms with varying T_1_ values were placed on a pneumatic motor that oscillated 20 mm in the antero-posterior direction every second. Animals: Under ACUC-approved protocol, six swine underwent 2 hr LAD occlusion. Imaging took place 4-25 weeks post-infarction and 10-3 min post double-dose injection of Magnevist (Schering, Germany). A 3D GRE sequence with 1.25x1.25x4 mm^3^ resolution and 2.5 min scan time ( assuming 100% gating efficiency) was used.

## Results

Figure 1 (c-j) compares PSIR and INPSIR. (c-d) images from moving agar gels demonstrate possible artifacts when no motion compensation is used for the reference images. Short-axis images from an infarcted swine model including reference image magnitude, single-coil phase and phase-sensitive reconstruction are shown. The phase reference image was improved with INPSIR (arrows) and navigator efficiency was not affected.

## Conclusions

INPSIR allows for improved reference images, uncorrupted by respiratory motion with no additional penalties in navigator efficiency. The lung-liver-heart boundaries were all sharper with INPSIR. We expect INPSIR will have a greater effect when using parallel imaging and may help obtain additional useful contrasts from phase reference images (5).

**Figure 2:**
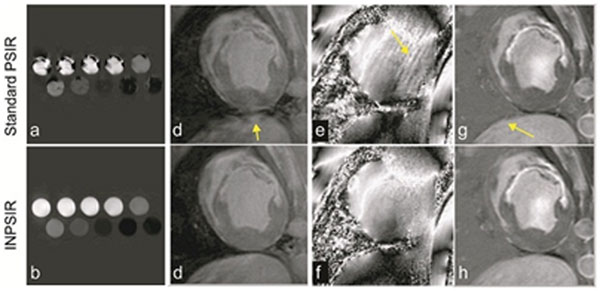
Comparison of images from PSIR and INPSIR: agar gels, reference image, single-coil phase reference, and phase-sensitive reconstructions. Note artifacts on phase image as well as lung-liver sharpness on INSPIR (arrows).

